# Impact of colonisation with multidrug-resistant gram-negative bacteria in a low endemicity setting: a population-based observational study

**DOI:** 10.1007/s15010-026-02878-9

**Published:** 2026-07-15

**Authors:** Vigith Andrews, Erik Senneby, Malin Inghammar, Oskar Ljungquist

**Affiliations:** 1 Clinical Microbiology, Infection Prevention and Control, Office for Medical Services, Region Skåne, 22185 Lund, Sweden; 2 Department of Infection Prevention and Control Region Skåne, Skåne University Hospital, 22185 Lund, Sweden; 3 Section for Infection Medicine, Department of Clinical Sciences Lund, Lund University, 22242 Lund, Sweden; 4 Clinical Microbiology, Department of Translational Medicine, Faculty of Medicine, Lund University, Malmö, Sweden; 5 Department of Infectious Diseases, Helsingborg Hospital, 25187 Helsingborg, Sweden

**Keywords:** Multidrug-resistant, Colonisation, Mortality, Morbidity, ESBL, Gram negative bacilli, Screening, Infection andprevention control

## Abstract

**Purpose:**

The health impact of multidrug-resistant gram-negative bacilli (MDR-GNB) colonisation in low-endemic settings remains insufficiently characterised. We aimed to evaluate mortality and healthcare utilisation associated with MDR-GNB colonisation in a Swedish population identified through targeted screening following universal risk assessment.

**Methods:**

All patients screened for MDR-GNB in Skåne, Sweden, between 2013 and 2022, were included, with one year of follow-up. Data on mortality (primary outcome), and secondary outcomes, including emergency department (ED) visits and hospital admissions, outpatient visits and antibiotic prescriptions, were extracted from health records. Logistic regression and Cox proportional hazards models were applied to compare patients with and without MDR-GNB colonisation, with odds ratio (OR) and hazard ratios (HR) quantifying associations.

**Results:**

Among 12,901 screened patients, 2,485 (19%) had colonisation with extended-spectrum β-lactamase–producing Enterobacterales, 83 (0.6%) with carbapenemase-producing *Enterobacterales*, 50 (0.4%) with carbapenem-resistant *Acinetobacter* spp. and 21 (0.2%) with carbapenem-resistant *Pseudomonas aeruginosa*. The overall one-year mortality was 6.6%, with no association between MDR-GNB colonisation and mortality (HR 0.96, 95% CI 0.80–1.14). MDR-GNB colonisation was, however, associated with increased risk of ED visits and hospital admissions, longer hospital stays, ICU-admissions, increased use of ambulatory and primary care, and higher antibiotic prescribing. These associations were most pronounced within the first three months post-screening and declined thereafter.

**Conclusions:**

Although MDR-GNB colonisation did not increase one-year mortality risk, patients with colonisation experienced significantly increased healthcare use. Further research is warranted to optimise patient outcomes, including preventive measures, and healthcare resource utilisation in this population.

**Supplementary Information:**

The online version contains supplementary material available at https://doi.org/10.1007/s15010-026-02878-9.

## Introduction

 The alarming global increase in multidrug-resistant organisms (MDROs) poses a grave public health threat, leading to increased morbidity and mortality rates [1, 2]. The World Health Organization classifies several gram-negative bacilli (GNB) among the highest priority pathogens: extended-spectrum β-lactamase-producing *Enterobacterales* (ESBL-E), carbapenemase-producing *Enterobacterales* (CPE), and carbapenem-resistant *Acinetobacter baumannii* are in the critical priority group, whereas carbapenem-resistant *Pseudomonas aeruginosa* (CRPA) is in the high priority category [3]. These pathogens can progress from colonisation to severe infections, even in immunocompetent individuals, and their extensive antimicrobial resistance profiles severely limit treatment options [4]. Their capacity for rapid dissemination, particularly within healthcare settings, under antimicrobial selective pressure further facilitates their competitive advantage [5].

Sweden remains a low-endemic country with historically low antibiotic resistance levels and a community carriage rate of 4.6% for ESBL-E [6]. However, recent increases in the incidence of ESBL-E (118 new cases per 100,000 inhabitants) and CPE (3.9 per 100,000 inhabitants) were documented in 2024 [7]. The National Infection Prevention and Control (IPC) guidelines introduced in the early 2000s mandate risk-based screening for notifiable MDRO like methicillin-resistant *Staphylococcus aureus* (MRSA), vancomycin resistant enterococci (VRE), and MDR-GNB like ESBL-E, CPE, CRPA and carbapenem-resistant *Acinetobacter* species (CRA) detection to prevent transmission within healthcare facilities and into the community. Healthcare facilities initiate contact precautions during screening and once a positive MDRO result is detected, these measures are upheld per guidelines. For CPE, contact precautions are continued during all future hospitalisations.

Most studies assessing the patient outcome of MDR-GNB have focused predominantly on invasive infections, high-risk patients and hospital wards, or short-term outcomes [8–10]. Current knowledge on the broader clinical consequences of MDR-GNB colonisation particularly in low-endemic settings remains limited [11–13]. Furthermore, a substantial knowledge gap remains regarding both short- and long-term post-discharge clinical outcomes, including mortality and healthcare use across broader population-based healthcare systems [14, 15]. Understanding these outcomes is essential for guiding evidence-based resource allocation and improving patient management while curbing further MDRO transmission.

In this study, the aim was to investigate the clinical burden associated with MDR-GNB colonisation, as measured by mortality rates, healthcare use across care levels, and antibiotic treatment following screening for MDR-GNB.

## Methods

### Study type and setting

We conducted a retrospective observational population-based cohort study including all patients screened for MDR-GNB across both community and hospital healthcare settings in Skåne, southern Sweden, a region with approximately 1.4 million inhabitants. The study period was October 1, 2013, to December 31, 2022, with a one-year follow-up for all patients. All patients who were hospitalised or received advanced outpatient care underwent mandatory universal risk assessment, combined with risk-based screening, according to local IPC guidelines. Screening was indicated if, within the past six months, the patient had: (1) been hospitalised or received advanced outpatient care outside the Nordic countries; (2) received care in a hospital or unit within the Nordic countries experiencing an ongoing MDRO outbreak; (3) spent more than one month outside the Nordic countries; or (4) worked in healthcare setting outside of Nordic countries. Community-based screening was also indicated in rehabilitation or long-term care facilities as part of outbreak management for residents exposed to MDR-GNB. All patients who underwent screening constituted the study population. The primary outcomes were 30-day and one-year all-cause mortality from the date of screening. The secondary outcomes included (1) acute care use (ED visit or any hospital admission), (2) length of hospitalisation, (3) intensive care unit (ICU) care, (4) outpatient healthcare use, defined as visits in specialised ambulatory and primary care, and (5) prescription of antibiotics. All secondary outcomes were analysed within the year post-screening.

Finally, the analyses of healthcare use were restricted to contacts in which patients received an infection-related diagnosis, either as the primary or secondary diagnosis according to the reclassification of the International Classification of Disease version 10 (ICD-10) established in a prior study (Supplementary Information, Table 1) [16].

### Data collection

Data on all screening cultures, regardless of growth along with sex, age, screening date, sample type (screening/clinical sample), and culture results, including species, antimicrobial susceptibility and resistance classification, were extracted from the databases of the sole Department of Clinical Microbiology serving all healthcare facilities in the Region Skåne, including long-term care, primary care, and hospitals. Screening samples were obtained from the rectum and, when relevant, from urine, wounds, tracheal samples, or indwelling devices. All clinical MDR-GNB isolates identified within one-year post-screening were reviewed for timing relative to the index screening. Using unique personal identification numbers, clinical information, including deaths, hospital admissions and discharges, public outpatient healthcare visits in specialised ambulatory care and primary care, and prescriptions was retrieved from electronic healthcare records. Comorbidities based on ICD-10 codes registered across all healthcare facilities within five years prior to the screening date were extracted from the Regional Healthcare Databases to calculate the Elixhauser comorbidity index (Supplementary Information, Table 2). Patients with known MDR-GNB carriage before the study period and those screened postmortem, were excluded. Samples from children < 1 year of age in neonatal units were also excluded, where local IPC routines stipulate weekly screening.

### Laboratory methods

The samples were cultured on chromogenic and selective agar. Species identification was performed using MALDI-TOF MS (Bruker Daltonics, Bremen Germany). Antimicrobial susceptibility testing was routinely conducted using disk diffusion and E-test methods, which were interpreted according to the European Committee on Antimicrobial Susceptibility Testing (EUCAST) breakpoints (http://www.eucast.org). Isolates exhibiting distinct phenotypic susceptibility profiles were considered unique. ESBL production in *Enterobacterales* isolates was tested for using synergy testing with inhibitor discs (AmpC & ESBL Detection Set, Mast Group). Carbapenem resistance was assessed locally and carbapenemase producing isolates were submitted to the Public Health Agency of Sweden following national surveillance protocols.

### Definitions

MDR-GNB was defined as ESBL-E, CPE, CRA, or CRPA. *Enterobacterales* producing both ESBL and carbapenemase were classified as CPE. Patients were considered carriers of MDR-GNB if their screening sample tested positive for any MDR-GNB; all others were considered non-carriers. The baseline was defined as the date of MDR-GNB screening, corresponding to the first positive screening for carriers and the first screening episode for non-carriers, with each patient included only once.

Screening samples were defined as specimens collected specifically to detect MDR-GNB carriage, whereas clinical samples were defined as specimens collected to investigate a suspected infection. Clinical infections were defined as growth with MDR-GNB in clinical samples.

### Analysis

Follow-up started on the date of screening and ended on the date of the outcome of interest, emigration, or after one year, whichever came first. The primary and secondary outcomes were estimated using Cox models and the proportional hazards assumption was evaluated with the Schoenfeld residuals test. The secondary outcomes were examined over two intervals of follow-up using landmark analyses [17]. To increase statistical power, two time periods (1–3 months and 4–12 months) were selected instead of quarterly intervals.

The length of hospital admission was analysed using linear regression, whereas the need of ICU care within one-year post-screening (yes or no) was analysed using logistic regression. Follow-up for all secondary outcomes commenced one week after discharge from hospital admission during which the index MDR-GNB screening was obtained. This washout period minimised readmissions due to nosocomial complications or factors directly related to the screening episode. Before a subsequent screening could be counted as a new episode, a minimum of one week post-discharge had to elapse.

The covariates used in all analyses were age, sex and MDR-GNB status. Age and sex were selected as established epidemiological risk factors for mortality and adverse healthcare outcomes. Age was included as a categorical covariate and was divided into four groups (1–26; 27–45; 46–67; and 68–105 years) to account for non-linear effects. The Elixhauser comorbidity index (ECI) encompasses 31 acute and chronic comorbidities (Supplementary Information, Table 2) [18]. The van Walraven score (VWS), a weighted ECI, was used to adjust for underlying baseline comorbidity burden and categorised into four groups (< 1, 1–5; 6–10; and > 10). Comorbidities not captured by ECI that differed significantly between patients with and without MDR-GNB carriage and may confound the association were additionally adjusted for. Accordingly, urinary tract diseases, defined by ICD-10 codes listed in Supplementary Information, Table 5, were included. Incidence of clinical MDR-GNB infections during follow-up was investigated using 1-Kaplan-Meier curves.

Data was analysed in R version 2024.12.0 + 467 (R: The R Project for Statistical Computing).

## Results

### Screening characterisation

**Fig. 1:**
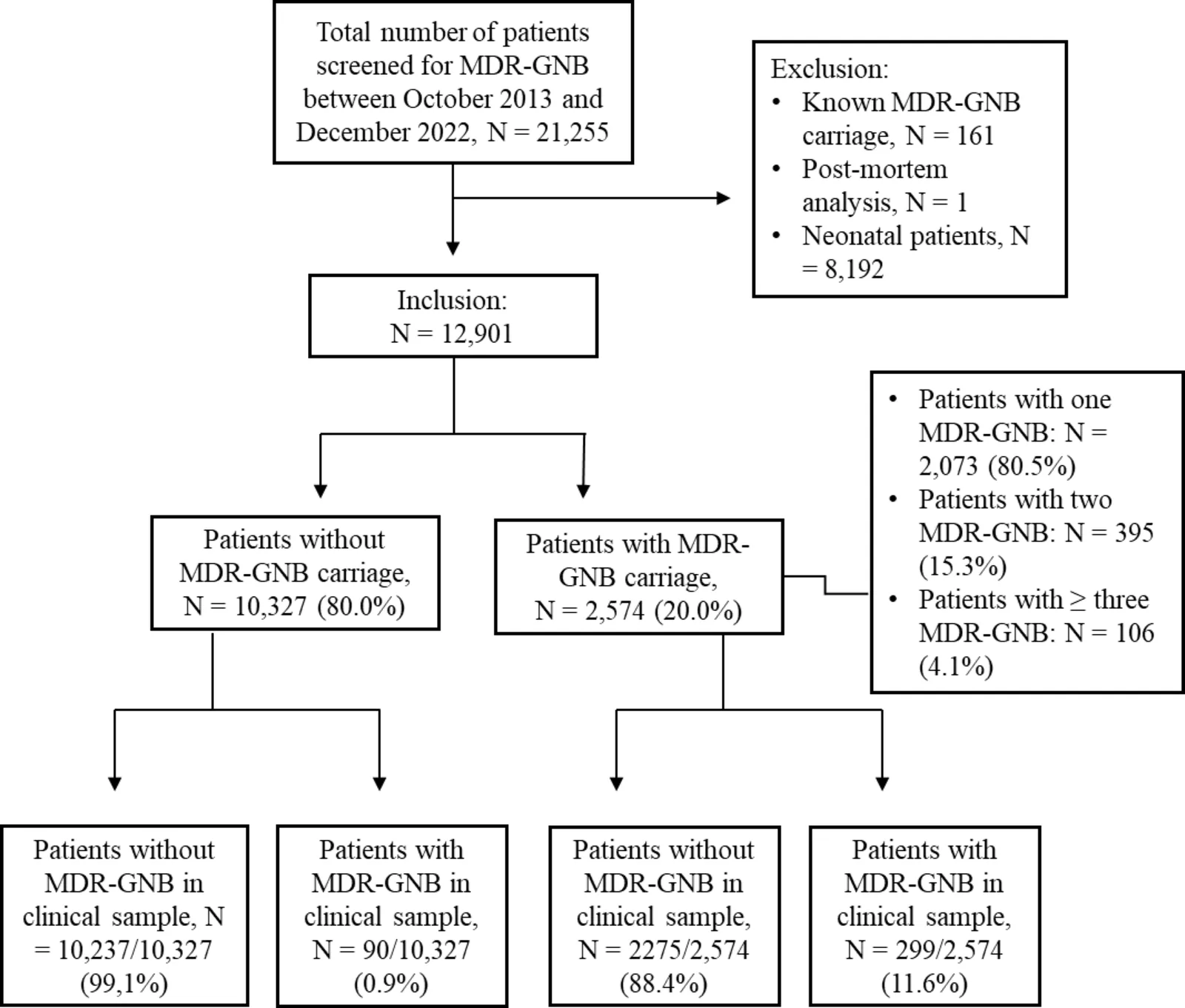
Study flowchart of patients included in the screening of multidrug-resistant gram-negative bacilli. Abbreviation: MDR-GNB, multidrug-resistant gram-negative bacilli

During the study period, 17,293 screening samples from 12,901 patients were included. Of these samples, 2842 (16.4%) yielded at least one MDR-GNB, corresponding to 2574 (20.0%) patients with carriage (Fig. 1).

Most of the samples originated from acute care hospital settings, including ED (23.5%) and internal medicine units (23.6%). Substantial screening also occurred in outpatient settings, such as ambulatory care (23.9%) and primary care clinics (14.6%), extending coverage to community-based facilities including long-term care institutions.

### Patient characterisation

The baseline comorbidity burden was low and comparable between patients with and without MDR-GNB carriage, with a median VWS of 0 (IQR 0–5) in both groups (*p* = 0.5) (Table 1).

**Table 1:** Baseline characteristics of included patients

Characteristic	Patients without MDR-GNB carriage (*n* = 10,327)	Patients with MDR-GNB carriage (*n* = 2,574)	*p*-value^1^
Age, years, median (Q1-Q3^2^)	46 (26–67)	43 (26–65)	0.036
Male, n (%)	4,878 (47.2%)	1,202 (46.7%)	0.6
Setting, n (%)			0.237
Inpatient	4,057 (39.3%)	1,044 (40.6%)	
Outpatient	6,245 (60.5%)	1,529 (59.4%)	
Other	25 (0.2%)	1 (0.0%)	
Van Walraven score, median (Q1-Q3^2^)	0 (0–5)	0 (0–5)	0.5
Van Walraven score, n (%)			0.209
< 1	6,645 (64.3%)	1,662 (64.6%)	
1–5	1,537 (14.9%)	388 (15.1%)	
6–10	899 (8.7%)	246 (9.6%)	
> 10	1,246 (12.1%)	278 (10.8%)	
Comorbidity, n (%)			
Dementia	143 (1.4%)	30 (1.2%)	0.4
Immunosuppression	1,349 (13.1%)	366 (14.2%)	0.13
Chronic kidney failure	607 (5.9%)	154 (6.0%)	0.9
Liver disease	228 (2.2%)	72 (2.8%)	0.089
Obesity	740 (7.2%)	216 (8.4%)	0.037
Chronic gastrointestinal diseases	429 (4.2%)	111 (4.3%)	0.8
Diseases of the urinary tract and reproductive organ	2,816 (27.3%)	784 (30.5%)	0.001
Cardiovascular disease	1,790 (17.3%)	367 (14.3%)	< 0.001
Peptic ulcer	47 (0.5%)	18 (0.7%)	0.2
Hypertension	2,493 (24.1%)	592 (23.0%)	0.2
Neurological disease	613 (5.9%)	171 (6.6%)	0.2
Chronic pulmonary diseases	1,063 (10.3%)	268 (10.4%)	0.9
Diabetes mellitus	1,140 (11.0%)	370 (14.4%)	< 0.001
Malignancy	1,033 (10.0%)	221 (8.6%)	0.033
Prior antibiotic use (2 weeks), n (%)	342 (3.3%)	94 (3.7%)	0.4
Prior peptic ulcer medication (2 weeks), n (%)	1,053 (10.2%)	267 (10.4%)	0.8

^1^Wilcoxon rank sum test; Pearson’s Chi-squared test

^2^Q1, first quartile; Q3, third quartile

Abbreviations: MDR-GNB, multidrug-resistant gram-negative bacilli. IQR, interquartile range

Overall, the comorbidity patterns were comparable; however, patients with MDR-GNB carriage presented a higher frequency of urinary tract diseases (31% versus 27%, *p* = 0.001), diabetes mellitus (14% versus 11%, *p* < 0.001) and obesity (8.4% versus 7.2%, *p* = 0.04), while being younger with a lower frequency of cardiovascular disease (14% vs. 17%, *p* < 0.001) and malignancy (8.6% versus 10.0%, *p* = 0.03).

### Frequency of MDR-GNB in carriers

The frequencies of carriage were 19.3% (2,485/12,901) for ESBL-E, 0.6% (83/12,901) for CPE, 0.4% (50/12,901) for CRA and 0.2% (21/12,901) for CRPA. Among the 2,574 patients with MDR-GNB carriage, 80.5% (*n* = 2073) harboured a single MDR-GNB species, while 15.3% (*n* = 395) and 4.1% (*n* = 106) also had two and three or more species, respectively (Fig. 1).

### Incidence of clinical infections

**Fig. 2:**
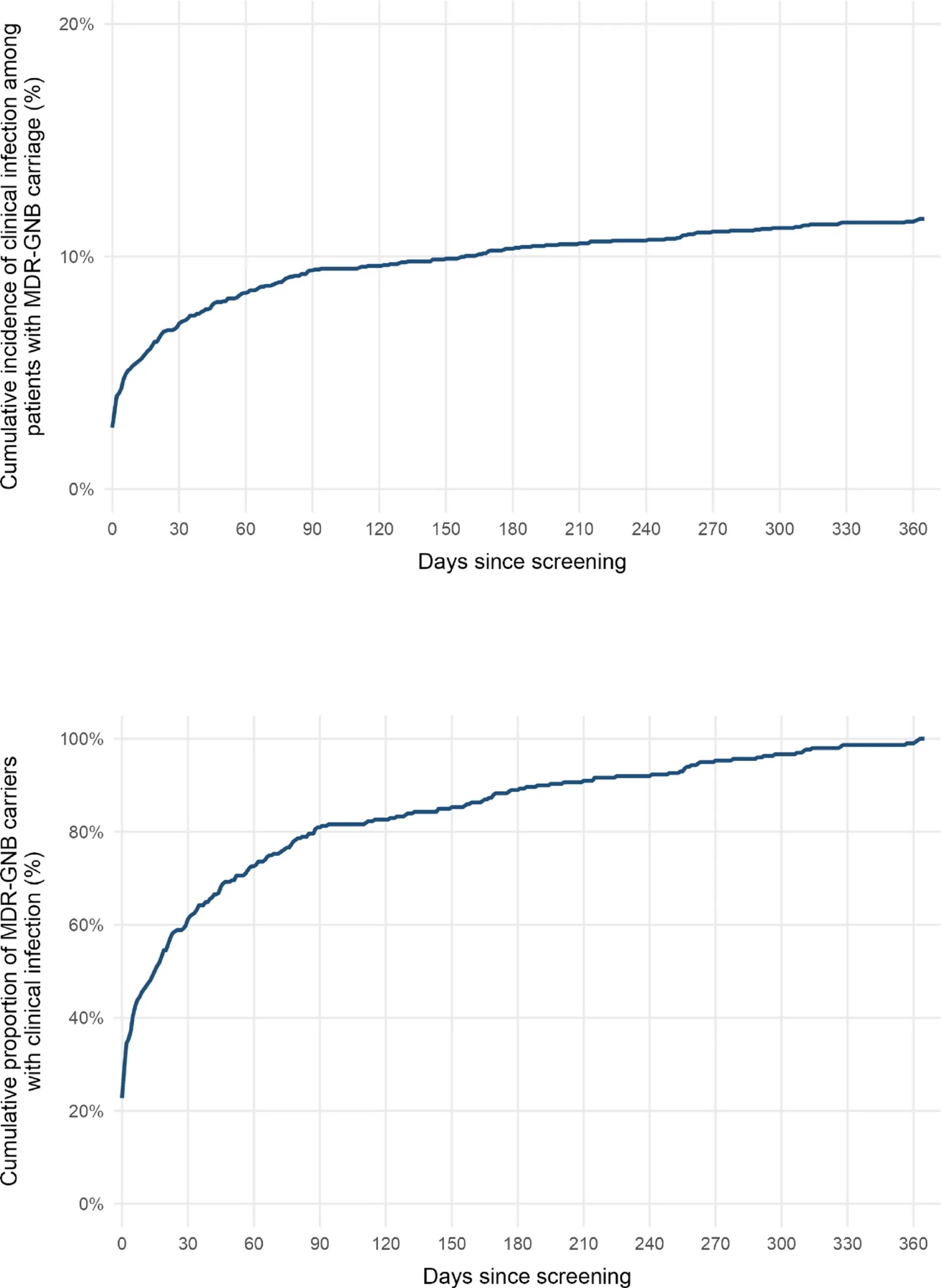
Risk and timing of clinical infection after MDR-GNB carriage. (a) Cumulative incidence of clinical infection among all patients with MDR-GNB carriage within 365 days after screening. (b) Cumulative proportion of infections occurring over time among patients who developed clinical infection. Abbreviation: MDR-GNB, multidrug-resistant gram-negative bacilli

During the year following screening, the cumulative incidence of clinical MDR-GNB infection among patients with MDR-GNB carriage was 11.6% (299/2574) compared with 0.9% (90/10,327) among patients without MDR-GNB carriage (Fig. 2).

Of the infections in patients with MDR-GNB carriage, 22.7% (68/299) were already present at the time of screening (day 0). The median time to clinical infection was 15 days (IQR 1–68) with the majority occurring within the first three months post-screening.

### Primary outcome

The overall one-year mortality risk in the cohort was 6.6%. The crude all-cause mortality rates at 30-day and one-year were 1.6% (*n* = 42) and 5.9% (*n* = 158), respectively, in patients with MDR-GNB carriage, compared to 1.7% (*n* = 172) and 6.8% (*n* = 699) respectively, in patients without carriage. Overall, mortality was higher among inpatients than outpatients, whereas crude mortality rates within each setting were similar regardless of carriage status (Supplementary Information, Table 6). In multivariable Cox regression model, MDR-GNB carriage was not associated with 30-day (HR 1.07, 95% CI 0.76–1.50) or one-year mortality (HR 0.96, 95% CI 0.80–1.14) (Table 2). Sensitivity analyses stratified by care setting and study period showed similar findings, with no significant associations in either inpatient or outpatient subgroups (Supplementary Information, Tables 6 and 7).

**Table 2:** Analyses of risk factors for 30-day and one-year mortality

Predictor	30-day mortality			One year mortality		
	Hazard Ratio (HR)	95% Confidence Interval	*p*-value	Hazard Ratio (HR)	95% Confidence Interval	*p*-value
Age, 1–26 years	Reference	-	-	Reference	-	-
Age, 27–45 years	1.15	0.40 - 3.29	0.791	2.23	1.37 - 3.61	0.001
Age, 46–67 years	5.77	2.62 - 12.72	< 0.001	5.81	3.83 - 8.83	< 0.001
Age, 68–105 years	9.01	4.11 - 19.73	< 0.001	10.77	7.13 - 16.25	< 0.001
Male sex	1.39	1.05 - 1.85	0.022	1.20	1.04 - 1.38	0.01
MDR-GNB carriage	1.07	0.76 - 1.50	0.692	0.96	0.80 - 1.14	0.622
Diseases of the urinary tract and reproductive organ	0.92	0.69 - 1.22	0.559	0.94	0.81 - 1.08	0.387
van Walraven score < 1	Reference	-	-	Reference	-	-
van Walraven score 1–5	1.82	1.10 - 3.02	0.02	2.51	1.97 - 3.20	< 0.001
van Walraven score 6–10	4.36	2.74 - 6.95	< 0.001	4.57	3.60 - 5.81	< 0.001
van Walraven score > 10	7.71	5.11 - 11.62	< 0.001	8.81	7.14 - 10.87	< 0.001

Abbreviation: MDR-GNB, multidrug-resistant gram-negative bacilli

Hazard ratios represent adjusted estimates from Cox regression models controlling for the predictors in the table

### Secondary outcomes

**Fig. 3:**
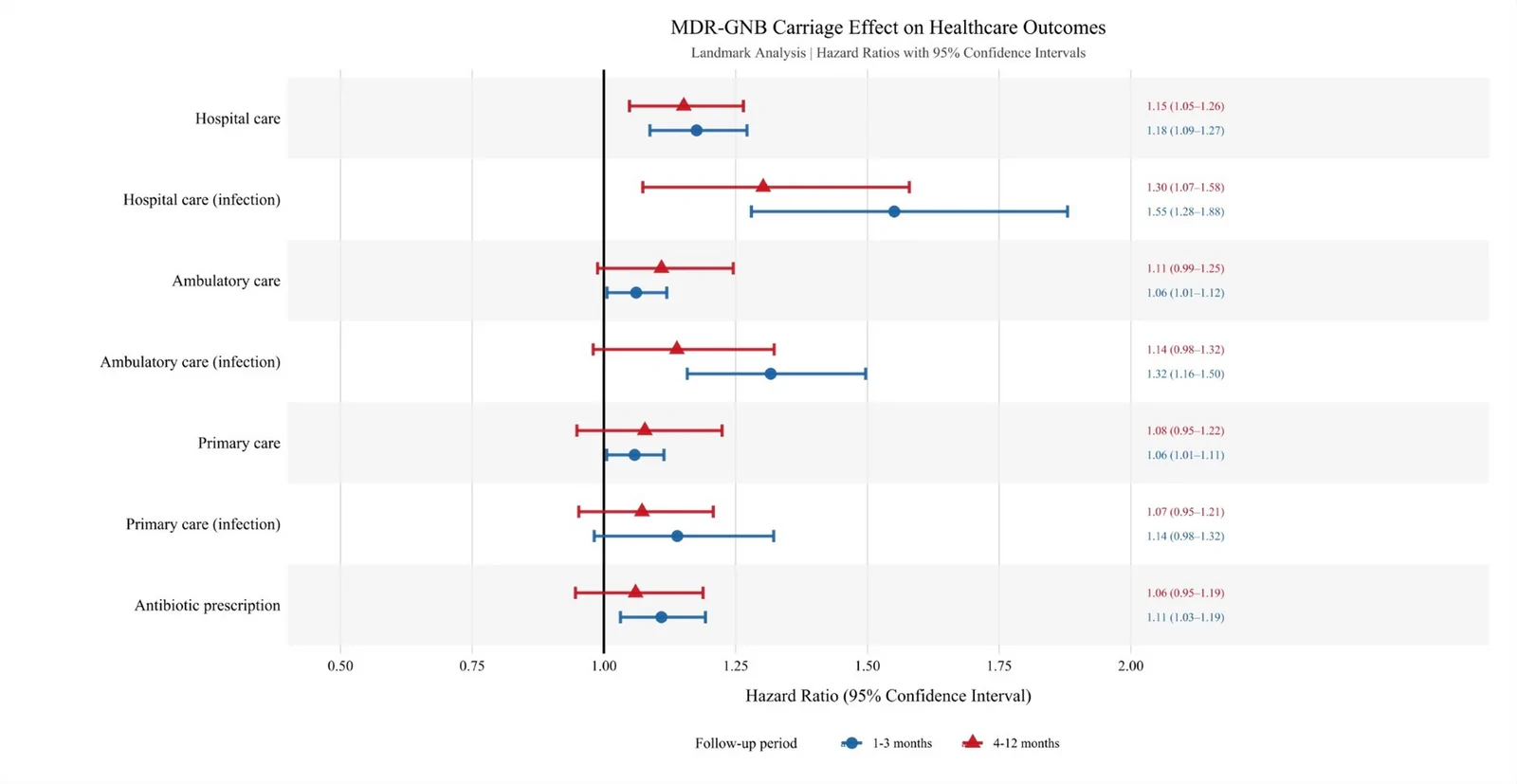
Landmark analysis of multidrug-resistant gram-negative bacilli carriage and its association with healthcare use. The blue circles denote estimates for the 1–3 month follow up interval, and the red circles denote estimates for the 4–12 month interval

Multivariable landmark analyses at three-month intervals revealed temporal variations in the association between MDR-GNB carriage and healthcare use during the year following screening (Fig. 3).

Overall, the risk of healthcare use increased during the first three months after screening and declined over time (data not shown); therefore, results for months 4–12 were aggregated. The strongest associations with MDR-GNB carriage were observed for ED visits and admissions (HR 1.18, 95% CI 1.09–1.27), infection-related ED visits and admissions (HR 1.55, 95% CI 1.28–1.88), and infection-related ambulatory care (HR 1.32, 95% CI 1.16–1.50) (Supplementary Information, Table 3). Sensitivity analyses stratified by inpatient versus outpatient status yielded results consistent with the primary analysis, with numerically stronger associations in outpatients (Supplementary Information, Fig. 1).

Within one-year post-screening, patients with MDR-GNB carriage had 1.8 additional hospital days (95% CI 0.82–2.84; *p* < 0.001), higher odds of ICU admission (OR 1.27, 95% CI 1.10–1.47; *p* < 0.001) when readmitted, and an increased risk of antibiotic prescription (HR 1.11, 95% CI 1.03–1.19; *p* = 0.005) compared with patients without carriage (Supplementary Information, Table 3).

## Discussion

In this large population-based study from a confined region in Sweden, we found that 20% of those who underwent universal risk-based screening were colonised with MDR-GNB, predominantly ESBL-E. During the first-year post-screening, 12% of patients with MDR-GNB carriage developed an infection confirmed by a positive clinical sample, with the majority occurring within the first 90 days. While ESBL-E colonisation is well known to predispose individuals to subsequent infections, the likelihood of developing infections varies considerably across healthcare settings and patient populations [19]. For example, infection rates of up to 26% have been reported among ICU patients. Furthermore, the risk of infection varies depending on the bacterial species, virulence factors and resistance mechanisms, with previous studies reporting rates ranging from 2 to 22% [15, 20–22].

The one-year mortality rate in patients with MDR-GNB carriage was low (5.9%) in our cohort, likely reflecting the substantial proportion of outpatients included. Although mortality was higher in inpatients than outpatients, setting-specific comparisons did not demonstrate a significant association between MDR-GNB carriage and mortality within either group. Likewise, in multivariate analyses, MDR-GNB carriage was not associated with increased all-cause mortality at either 30 days or one year. Although high mortality rates associated with MDR-GNB infections have been widely reported across both high- and low-endemic regions [23–25], the impact of colonisation alone remains less well established. A study from Taiwan examined all-cause mortality post-discharge in MDR-GNB screening positive patients over a 12-month follow-up period [14]. In addition to mortality associated with MDR-GNB infections, a significantly elevated risk for all-cause mortality after discharge was identified (HR, 2.29; 95% CI, 1.19–4.41) compared with screening-negative patients suggesting that colonisation may reflect underlying vulnerability. In contrast, several studies primarily originating from low-endemic, high-income countries have found minimal or no mortality differences, even among high-risk ICU patients with invasive infections with MDR-GNB [8, 9, 21, 26].

Unlike previous studies that have focused mainly on short term follow-up and mortality, our study also assessed the secondary burden of MDR-GNB colonisation over a full year, providing new insights from a low-endemic setting [9, 23, 27]. We found that colonisation was independently associated with increased healthcare use and a more prolonged healthcare burden than anticipated, as patients with MDR-GNB carriage demonstrated higher rates of hospital readmission and outpatient consultations throughout the one-year follow-up. Sensitivity analyses suggested that this association was consistent across care settings. The numerically stronger association observed among outpatients may reflect differences in case-mix, healthcare-seeking behaviour, or follow-up patterns, and suggests that outpatient carriers could represent an important group warranting further study.

The associations remained consistent during the first three months post-screening and were particularly pronounced for infection-related visits. This pattern is biologically plausible, as previous studies have shown that most MDR-GNB infections occur early, with more than half detected within three months of screening [14, 15]. Our findings indicate that MDR-GNB carriage may identify a subgroup at increased infection-related healthcare burden whether reflecting increased infection risk, greater treatment challenges, or unmeasured differences in healthcare exposure. Given that most MDR-GNB infections in our cohort occurred within three months of screening, these results support increased clinical vigilance for patients identified with MDR-GNB carriage. Moreover, with hospitalisation costs in Sweden estimated at €1,800 per day, and substantially higher for intensive care, a considerable economic burden on healthcare resources could arise from the additional two hospital days associated with MDR-GNB carriage [28].

The risk of antibiotic treatment was 11% higher in patients with MDR-GNB carriage throughout the one-year follow-up period, warranting further investigation into potential indication bias, as clinicians may have a lower threshold for prescribing antibiotics when patients are known to carry MDR-GNB. These patients may also have been at increased risk of acquiring MDR-GNB initially due to prior antibiotic exposure or other unmeasured risk factors, which could explain their continued elevated risk of receiving antibiotic treatment following detection of MDR-GNB carriage.

The main strength of this study lies in its population-based design, with patient characteristics extracted from comprehensive regional and national data registers. As the sole clinical microbiology laboratory in the region, we could systematically include all screened patients and capture all subsequent MDR-GNB detections across all healthcare institutions within the area.

Several limitations warrant consideration. We grouped ESBL-E, CPE, CRA and CRPA together in the analyses, however the absolute majority of MDR-GNB found was ESBL-E. The complete identification of all ESBL and carbapenemases was not feasible because of the absence of systematic local monitoring, although stratification of resistance mechanisms could have provided additional insight into associations with infections and mortality. Most importantly, this register-based study of more than 12,000 screened patients did not permit evaluation of specific healthcare exposures, such as residence in long-term care facilities, prior domestic or foreign hospitalisation in endemic areas, or international travel. These recognised risk factors for MDR-GNB colonisation could therefore not be analysed separately, leaving the possibility of residual confounding. In addition, despite restricting the comparison to screened patients only, patients with MDR-GNB carriage may differ in unmeasured case-mix or healthcare exposure profiles. The higher healthcare use observed in patients with carriage during follow-up may reflect such rather than measured comorbidity alone, given that overall comorbidity burden was comparable between carriers and non-carriers, as reflected by similar VWS, and was accounted for in the adjusted analyses. Furthermore, the association with increased healthcare use was observed in both inpatient and outpatient sensitivity analyses, suggesting that the findings were not solely explained by differences in initial care setting. Importantly, despite greater healthcare use, MDR-GNB carriage was not associated with increased all-cause mortality at either 30 days or one year. Nevertheless, the increased healthcare use suggests that MDR-GNB carriage may identify a subgroup with greater healthcare burden. These findings underscore the importance of increased vigilance in individuals identified with MDR-GNB carriage through risk-based screening, and further studies should evaluate targeted interventions to mitigate complications in this population.

## Conclusion

Carriage of multidrug-resistant gram-negative bacilli did not increase the mortality risk during the one-year follow-up period. However, patients with MDR-GNB carriage experienced increased morbidity especially during the first three months post-screening. These findings warrant further investigation to optimise patient outcomes, including preventive measures, and healthcare resource use for this population.

## Supplementary Information

Below is the link to the electronic supplementary material.

Supplementary Material 1

## Data Availability

Due to Swedish legal and ethical restrictions, the data underlying this study cannot be made publicly available. De-identified data may be shared upon reasonable request and subject to approval by the Swedish Ethical Review Authority.
