# Supplementary material for: Impact of colonisation with multidrug-resistant gram-negative bacteria in a low endemicity setting: a population-based observational study
Source: Infection. 2026 Jul 15;54(5):2539–48. doi: 10.1007/s15010-026-02878-9 (PMC13727845; doi:10.1007/s15010-026-02878-9)
Supplement: Supplementary file 1 [file 15010_2026_2878_MOESM1_ESM.docx]

Impact of colonisation with multidrug-resistant Gram-negative bacteria in a low endemicity setting: a population-based observational study.

Infections.

Andrews V, Senneby E, Inghammar M, Ljungquist O. Clinical Microbiology, Infection Prevention and Control, Office for Medical Services, Region Skåne. Sölvegatan 23B, 22185 Lund, Sweden (vigith.andrews@skane.se).

# Table 1. Diagnoses classified as infectious diseases, by ICD-10 chapter

| **ID category** | **Codes** |
| --- | --- |
| Enteric infections | A00 – A09 |
| Bloodstream infections | A40 – A41, R572, R650 – R651 |
| Sexually transmitted infections | A50 – A64, B20 – B24 |
| Infections of the neurological system, including the eye | A39, A80–A89, B30, G00 – G02, G039, G040 – G042, G049, G05–G08, G940, H000, H03, H043, H050, H061, H100, H102, H103, H109, H130, H131, H160, H162, H168, H169, H190 – H192, H220, H320, H440, H451 |
| Upper respiratory tract infections, including the ear | H600–H603, H608, H609, H610, H62, H66, H67, H70, H730, H750, H830, H940, J00 – J06, J340, J36, J390, J391 |
| Lower respiratory tract infections, including influenza | A15 – A19, A481, J09 – J22, J440 – J441, J690, J85 – J86 |
| Infections of heart and blood vessels | I301, I320, I321, I33, I38, I39, I400, I410 – I412, I430, I520, I521, I681, I790, I791, I980, I981 |
| Infections of the digestive system including the liver, narrow definition | B15 – B19, K044, K046, K047, K102, K113, K122, K230, K231, K61, K630, K650, K67, K750, K770, K830, K930, K931 |
| Infections of the genitourinary system | N080, N10, N136, N151, N159, N160, N290, N291, N300, N309, N330, N340, N341, N37, N390, N410, N412 – N413, N431, N450, N459, N481, N482, N49, N51, N61, N70 – N74, N751, N764, N770, N771 |
| Infections of skin and soft tissue | A46, L00 – L04, L050, L08, L303 |
| Infections of bone, joints and connective tissue | M00 – M01, M462 – M465, M490 – M492, M600, M630 – M632, M650, M651, M680, M710, M711, M726, M730, M731, M86, M900 – M902 |
| Infectious complications | T793, T802, T814, T826*, T827*, T835*, T836*, T845 – T847*, T857*, T874, T880 |
| Other infections | A20 – A28, A30 – A38, A42 – A49, A65 – A69, A70 – A74, A75 – A79, A92 – A99, B00 – B09, B25 – B27, B33 – B99, R508, R509, D709, D733, E060, E321 |
| Pregnancy, childbirth and the puerperium | O030, O035, O040, O045, O050, O055, O060, O065, O070, O075, O080, O23, O411, O753, O85, O86, O91, O98 |
| Diseases of the digestive system – wide definition | above + K251, K252, K255, K256, K261, K262, K265, K266, K271, K272, K275, K276, K281, K282, K285, K286, K35, K401, K404, K411, K414, K421, K431, K434, K437, K441, K451, K461, K550, K56, K57, K800 – K804, K810, K819 |

# Table 2. Diagnoses used for Elixhauser Comorbidity Index, by ICD-10 codes

**1. Congestive Heart Failure**

I09.9, I11.0, I13.0, I13.2, I25.5, I42.x, I43.x, I50.x, P29.0

**2. Cardiac Arrhythmias**

I44.x–I49.x, R00.0, R00.1, R00.8

**3. Valvular Disease**

I05.x–I08.x, I34.x–I37.x, I39.x, Q23.x

**4. Pulmonary Circulation Disorders**

I26.x–I28.x

**5. Peripheral Vascular Disorders**

I70.x–I79.x, I96, Z95.8, Z95.9

**6. Hypertension, Uncomplicated**

I10

**7. Hypertension, Complicated**

I11.x, I12.x, I13.x, I15.x (when linked to organ damage)

**8. Paralysis**

G04.1, G11.4, G80.x–G83.x

**9. Other Neurological Disorders**

G10.x–G14.x, G20.x–G26.x, G30.x–G32.x, G35.x–G37.x, G40.x–G47.x, R47.x, R56.x

**10. Chronic Pulmonary Disease**

J40–J47, J60–J67, J68.4, J70.1, J70.3

**11. Diabetes, Uncomplicated**

E10.9, E11.9, E13.9

**12. Diabetes, Complicated**

E08.x–E13.x **with** .2–.9 complications (renal, ophthalmic, neurologic, circulatory)

**13. Hypothyroidism**

E00.x–E03.x, E89.0

**14. Renal Failure**

I12.0, I13.1, N18.x, N19, N25.0, Z49.x, Z91.15, Z99.2

**15. Liver Disease**

K70.x, K71.x, K72.x, K73.x, K74.x, K75.2–K75.4, K75.9, K76.x

**16. Peptic Ulcer Disease (excluding bleeding)**

K27.9, Z87.11

**17. AIDS/HIV**

B20–B24

**18. Lymphoma**

C81.x–C85.x, C88.x, C96.x, C90.0

**19. Metastatic Cancer**

C77.x–C80.x

**20. Solid Tumor Without Metastasis**

C00.x–C76.x (excluding metastatic codes)

**21. Rheumatoid Arthritis / Collagen Vascular Diseases**

M05.x–M06.x, M08.x, M30.x–M36.x, D86.x

**22. Coagulopathy**

D65–D68.x, D69.1, D69.3, D69.5

**23. Obesity**

E66.x

**24. Weight Loss / Cachexia**

R63.4, R64, E43, E44.x

**25. Fluid and Electrolyte Disorders**

E22.2, E86.x, E87.x

**26. Blood Loss Anemia**

D50.0, D62

**27. Deficiency Anemia**

D50.8, D50.9, D51.x–D53.x

**28. Alcohol Abuse**

F10.x, E24.4, K70.1–K70.3, K70.9, Z50.2, Z71.4, Z72.1

**29. Drug Abuse**

F11.x–F16.x, F18.x, F19.x, Z50.3, Z71.5, Z72.2

**30. Psychoses**

F20.x–F29.x

**31. Depression**

F32.x–F33.x

# Table 3. Secondary outcomes, one-year follow-up

Hazard ratios from Cox regression models adjusted for age, sex, disease of the urinary tract and reproductive organs, and van Walraven score.

Corresponding results are illustrated in Figure 3.

## Hospital admission

| Time Period | Predictor Variable | Patients Analysed | Number of Events | HR (95% CI) | p-value |
| --- | --- | --- | --- | --- | --- |
| 0-90 days | MDR-GNB carriage | 12899 | 3655 | 1.18 (1.09-1.27) | <0.001 |
| 91-365 days | MDR-GNB carriage | 9011 | 2614 | 1.15 (1.05-1.26) | 0.003 |

## Hospital admission, infection related

| Time Period | Predictor Variable | Patients Analysed | Number of Events | HR (95% CI) | p-value |
| --- | --- | --- | --- | --- | --- |
| 0-90 days | MDR-GNB carriage | 12899 | 531 | 1.55 (1.28-1.88) | <0.001 |
| 91-365 days | MDR-GNB carriage | 11997 | 570 | 1.30 (1.07-1.58) | 0.008 |

## Ambulatory care

| Time Period | Predictor Variable | Patients Analysed | Number of Events | HR (95% CI) | p-value |
| --- | --- | --- | --- | --- | --- |
| 0-90 days | MDR-GNB carriage | 12899 | 8250 | 1.06 (1.01-1.12) | 0.026 |
| 91-365 days | MDR-GNB carriage | 4454 | 1759 | 1.11 (0.99-1.25) | 0.078 |

## Ambulatory care, infection related

| Time Period | Predictor Variable | Patients Analysed | Number of Events | HR (95% CI) | p-value |
| --- | --- | --- | --- | --- | --- |
| 0-90 days | MDR-GNB carriage | 12899 | 1268 | 1.32 (1.16-1.50) | <0.001 |
| 91-365 days | MDR-GNB carriage | 11262 | 1005 | 1.14 (0.98-1.32) | 0.088 |

## Primary care

| Time Period | Predictor Variable | Patients Analysed | Number of Events | HR (95% CI) | p-value |
| --- | --- | --- | --- | --- | --- |
| 0-90 days | MDR-GNB carriage | 12899 | 8979 | 1.06 (1.01-1.11) | 0.036 |
| 91-365 days | MDR-GNB carriage | 3658 | 1520 | 1.08 (0.95-1.22) | 0.228 |

## Primary care, infection related

| Time Period | Predictor Variable | Patients Analysed | Number of Events | HR (95% CI) | p-value |
| --- | --- | --- | --- | --- | --- |
| 0-90 days | MDR-GNB carriage | 12899 | 1006 | 1.14 (0.98-1.32) | 0.090 |
| 91-365 days | MDR-GNB carriage | 3658 | 1639 | 1.07 (0.95-1.21) | 0.270 |

## Antibiotic prescription

| Time Period | Predictor Variable | Patients Analysed | Number of Events | HR (95% CI) | p-value |
| --- | --- | --- | --- | --- | --- |
| 0-90 days | MDR-GNB carriage | 12576 | 4376 | 1.11 (1.03-1.19) | 0.005 |
| 91-365 days | MDR-GNB carriage | 8041 | 1853 | 1.06 (0.95-1.19) | 0.310 |

## Length of admission

| Predictor | Beta (95% CI) for Admission Length (days) | p-value |
| --- | --- | --- |
| Age | 0.02 (0.002 - 0.039) | 0.032 |
| Male sex | 0.875 (0.055 - 1.70) | 0.037 |
| MDR-GNB carriage | 1.83 (0.818 - 2.84) | <0.001 |
| Diseases of the urinary tract | 1.44 (0.465 - 2.42) | 0.004 |
| van Walraven score 1-5 | 4.33 (3.12 - 5.54  ) | <0.001 |
| van Walraven score 6-10 | 10.1 (8.60 - 11.6) | <0.001 |
| van Walraven score >10 | 13.9 (12.5 - 15.3) | <0.001 |

Admission days were estimated using linear regression models adjusted for the predictors in the table.

## ICU care

| Predictor | OR (95% CI) for ICU care | p-value |
| --- | --- | --- |
| Age | 0.997 (0.994 - 1) | 0.03 |
| Male sex | 1.12 (0.991 - 1.27) | 0.07 |
| MDR-GNB carriage | 1.27 (1.10 - 1.47) | <0.001 |
| Diseases of the urinary tract | 1.63 (1.42 - 1.87) | <0.001 |
| van Walraven score 1-5 | 1.76 (1.48 - 2.10) | <0.001 |
| van Walraven score 6-10 | 2.23 (1.83 - 2.70) | <0.001 |
| van Walraven score >10 | 2.40 (1.99 - 2.88) | <0.001 |

Odds ratios (OR) from logistic regression models adjusted for the predictors in the table.

# Table 4. Distribution of MDR-GNB in screening samples from 2574 carriers.

| Species | Number of isolates | Number of patients |
| --- | --- | --- |
| *Escherichia coli* | 2702 | 2354 |
| - EPE | 2644 | 2325 |
| - CPE | 53 | 43 |
| *Klebsiella pneumoniae* | 399 | 373 |
| - EPE | 335 | 321 |
| - CPE | 61 | 51 |
| *Proteus mirabilis* | 26 | 24 |
| - EPE | 23 | 23 |
| - CPE | 3 | 3 |
| *Salmonella* species | 3 | 3 |
| - EPE | 3 | 3 |
| - CPE | 0 | 0 |
| *Klebsiella aerogenes* | 3 | 3 |
| *Enterobacter cloacae* | 2 | 2 |
| Other *Enterobacterales* |  |  |
| *Acinetobacter* species | 57 | 50 |
| *Pseudomonas aeruginosa* | 24 | 21 |

# Table 5. Diagnoses classified as diseases of the urinary tract and reproductive organ, by ICD-10 chapter

| **Disease category** | **Codes** |
| --- | --- |
| Glomerular diseases | N00.0–N08.9 |
| Renal tubulo-interstitial diseases and renal failure | N10.0–N19.9 |
| Urolithiasis (kidney and urinary tract stones) | N20.0–N20.9 |
| Other disorders of kidney and ureter | N25.0–N29.9 |
| Other diseases of the urinary system | N30.0–N39.9 |
| Diseases of male genital organs (including prostate disorders and prostatitis) | N40.0–N41.9 |

# Table 6. Inpatient versus outpatient mortality

## Crude mortality rates

| **Setting** | **Outcome** | **Patients without MDR-GNB carriage** | **Patients with MDR-GNB carriage** | **p-value** |
| --- | --- | --- | --- | --- |
| Inpatient | 30-day mortality | 135/4057 (3.3%) | 32/1044 (3.1%) | 0.671 |
|  | 1-year mortality | 438/4057 (10.8%) | 86/1044 (8.2%) | 0.015 |
| Outpatient | 30-day mortality | 36/6245 (0.6%) | 10/1529 (0.7%) | 0.723 |
|  | 1-year mortality | 256/6245 (4.1%) | 66/1529 (4.3%) | 0.702 |

Abbreviation: MDR-GNB, multidrug-resistant gram-negative bacilli.

## Cox regression for 30-day mortality

Hazard ratios represent adjusted estimates from Cox regression models controlling for the predictors in the table. Age was modelled as a continuous variable to preserve statistical power given the smaller sample size.

|  | Outpatient | | | Inpatient | | |
| --- | --- | --- | --- | --- | --- | --- |
| Predictor | Hazard Ratio (HR) | 95% Confidence Interval | p value | Hazard Ratio (HR) | 95% Confidence Interval | p value |
| Age (per year) | 1.06 | 1.04 – 1.08 | <0.001 | 1.03 | 1.02 – 1.04 | <0.001 |
| Male sex | 1.64 | 0.89 - 3.02 | 0.116 | 1.36 | 0.98 -1.87 | 0.062 |
| MDR-GNB carriage | 1.48 | 0.73 - 3 | 0.28 | 0.98 | 0.67- 1.45 | 0.934 |
| Diseases of the urinary tract | 0.68 | 0.36 - 1.26 | 0.217 | 0.94 | 0.68-1.29 | 0.695 |
| van Walraven score <1 | Reference | - | - | Reference | - | - |
| Van Walraven score >1-5 | 1.8 | 0.65 – 4.98 | 0.258 | 1.41 | 0.79-2.53 | 0.247 |
| Van Walraven score 6-10 | 1.42 | 0.37 – 5.42 | 0.606 | 3.37 | 2.01-5.64 | <0.001 |
| van Walraven score >10 | 9.7 | 4.19 – 22.47 | <0.001 | 4.31 | 2.68-6.92 | <0.001 |

## Cox regression for one-year mortality

|  | Outpatient | | | Inpatient | | |
| --- | --- | --- | --- | --- | --- | --- |
| Predictor | Hazard Ratio (HR) | 95% Confidence Interval | p value | Hazard Ratio (HR) | 95% Confidence Interval | p value |
| Age (per year) | 1.05 | 1.04-1.06 | <0.001 | 1.03 | 1.03-.1.04 | <0.001 |
| Male sex | 1.3 | 1.04-1.63 | 0.02 | 1.23 | 1.03-1.47 | 0.022 |
| MDR-GNB carriage | 1.31 | 0.99-1.72 | 0.055 | 0.82 | 0.65-1.04 | 0.098 |
| Diseases of the urinary tract | 0.81 | 0.64-1.03 | 0.084 | 0.96 | 0.8-1.15 | 0.659 |
| van Walraven score <1 | Reference | - | - | Reference | - | - |
| Van Walraven score >1-5 | 2.19 | 1.52-3.17 | <0.001 | 2.05 | 1.5-2.82 | <0.001 |
| Van Walraven score 6-10 | 4.3 | 2.97-6.23 | <0.001 | 3.13 | 2.29-4.29 | <0.001 |
| van Walraven score >10 | 8.16 | 5.88-11.33 | <0.001 | 5.69 | 4.32-7.49 | <0.001 |

# Table 7. Mortality analysis using a Cox model stratified by study period

Mortality was analysed using a stratified Cox proportional hazards model, with calendar study period (October 2013–2016, 2017–2019, and 2020–2022) as the stratification variable.

Hazard ratios represent adjusted estimates from Cox regression models controlling for the predictors in the table.

|  | 30-day mortality | | | One year mortality | | |
| --- | --- | --- | --- | --- | --- | --- |
| Predictor | Hazard Ratio (HR) | 95% Confidence Interval | p-value | Hazard Ratio (HR) | 95% Confidence Interval | p-value |
| Age, 1-26 years | Reference | - | - | Reference | - | - |
| Age, 27-45 years | 1.16 | 0.41–3.31 | 0.784 | 2.21 | 1.36–3.58 | 0.001 |
| Age, 46-67 years | 5.76 | 2.61–12.70 | <0.001 | 5.73 | 3.77–8.70 | <0.001 |
| Age, 68-105 years | 8.90 | 4.06–19.51 | <0.001 | 10.57 | 7.01–15.96 | <0.001 |
| Male sex | 1.40 | 1.05–1.86 | 0.021 | 1.21 | 1.05–1.39 | 0.008 |
| MDR-GNB carriage | 1.06 | 0.76–1.49 | 0.724 | 0.96 | 0.81–1.15 | 0.689 |
| Diseases of the urinary tract and reproductive organ | 0.93 | 0.70–1.24 | 0.640 | 0.96 (0.83–1.11) | 0.83–1.11 | 0.592 |
| van Walraven score <1 | Reference | - | - | Reference | - | - |
| van Walraven score 1-5 | 1.82 | 1.09–3.02 | 0.021 | 2.51 | 1.97–3.19 | <0.001 |
| van Walraven score 6-10 | 4.34 | 2.72–6.92 | <0.001 | 4.58 | 3.60–5.81 | <0.001 |
| van Walraven score >10 | 7.63 | 5.06–11.50 | <0.001 | 8.83 | 7.16–10.89 | <0.001 |

Abbreviation: MDR-GNB, multidrug-resistant gram-negative bacilli.

Hazard ratios represent adjusted estimates from Cox regression models controlling for the predictors in the table.

# Figure 1. Sensitivity analyses of secondary outcomes stratified by inpatient versus outpatient status

Landmark analysis of multidrug-resistant gram-negative bacilli carriage and its association with healthcare utilisation. The blue circles denote estimates for the 1–3 month follow- up interval, and the red circles denote estimates for the 4–12 month interval).

# Figure 2. Temporal trends in one-year mortality and cohort size by MDR-GNB carriage status
